# Transcriptome-Wide Identification and Characterization of Potato Circular RNAs in Response to *Pectobacterium carotovorum* Subspecies *brasiliense* Infection

**DOI:** 10.3390/ijms19010071

**Published:** 2017-12-27

**Authors:** Ran Zhou, Yongxing Zhu, Jiao Zhao, Zhengwu Fang, Shuping Wang, Junliang Yin, Zhaohui Chu, Dongfang Ma

**Affiliations:** 1 College of Agriculture/College of Horticulture and Gardening/Hubei Key Laboratory of Waterlogging Disaster and Agricultural Use of Wetland, Yangtze University, Jingzhou 434025, China; sgrsmart@nwafu.edu.cn (R.Z.); yongxingzhu@yangtzeu.edu.cn (Y.Z.); 2011060035@nwafu.edu.cn (J.Z.); zhengwufang@yangtzeu.edu.cn (Z.F.); wangshuping@yangtzeu.edu.cn (S.W.); 2 College of Agronomy, Shandong Agricultural University, Tai’an 271018, China; 3 College of Plant Protection, Northwest A&F University, Yangling 712100, China

**Keywords:** potato circRNAs, *Pectobacterium carotovorum*, parental genes, miRNA sponges, GO enrichment, WGCNA

## Abstract

Little information about the roles of circular RNAs (circRNAs) during potato-*Pectobacterium carotovorum* subsp. *brasiliense* (*Pcb*) interaction is currently available. In this study, we conducted the systematic identification of circRNAs from time series samples of potato cultivars Valor (susceptible) and BP1 (disease tolerant) infected by *Pcb*. A total of 2098 circRNAs were detected and about half (931, 44.38%) were intergenic circRNAs. And differential expression analysis detected 429 significantly regulated circRNAs. circRNAs play roles by regulating parental genes and sponging miRNAs. Gene Ontology (GO) enrichment of parental genes and miRNAs targeted mRNAs revealed that these differentially expressed (DE) circRNAs were involved in defense response (GO:0006952), cell wall (GO:0005199), ADP binding (GO:0043531), phosphorylation (GO:0016310), and kinase activity (GO:0016301), suggesting the roles of circRNAs in regulating potato immune response. Furthermore, weighted gene co-expression network analysis (WGCNA) found that circRNAs were closely related with coding-genes and long intergenic noncoding RNAs (lincRNAs). And together they were cultivar-specifically regulated to strengthen immune response of potato to *Pcb* infection, implying the roles of circRNAs in reprogramming disease responsive transcriptome. Our results will provide new insights into the potato-*Pcb* interaction and may lead to novel disease control strategy in the future.

## 1. Introduction

Circular RNAs (circRNAs) have recently become the intensive study object in a wide variety of organisms [1]. Different from the traditional linear RNAs, circRNAs lack 5’ and 3’ ends and usually are generated by head-to-tail backsplicing events [2]. Its observation was firstly described as rare events, nevertheless, in recent years, increasing evidence suggests that circRNAs are widely existent in eukaryotic cells and commonly produced by thousands of genes [3].

One interesting research area is circRNAs’ biological roles in transcriptional and post-transcriptional regulation. Recent studies suggested that circRNAs can function as microRNA (miRNA) sponges, as transcription *cis*-regulators, as RNAs processing intermediates, as snoRNAs, and so on [3]. The most well studied function of circRNAs is acting like sponges to soak up miRNAs. The best known example is *CDR1as*, which contains 63 potential binding sites for *miR*-*7* and can negatively regulate the function of *miR*-*7* in humans [4]. Another prominent function of circRNAs is to regulate gene expression. For example, Zhang et al. [2] reported that knockdown of intronic circRNAs led to the reduced expression of their parental genes. Conversely, exon-intron circRNAs, another class of the circRNAs, have been reported to enhance the expression of their parental genes via specific RNA-RNA interaction as *cis*-regulators. Besides, recent studies suggested that some circRNAs can regulate cell-cycle by interacting with proteins or even function as mRNAs to directly synthesize proteins [4,5,6]. However, the biological functions of most animal circRNAs are largely unknown currently [7].

Compared with animals, much less information about the biogenesis, regulation, and function of circRNAs is known in plants [8]. Wang et al. [9] first discovered circRNAs in *Arabidopsis thaliana*. Then Ye et al. [10] identified substantial circRNAs in *A*. *thaliana* and *Oryza sativa* through genome-wide identification, and proposed that plants and animals circRNAs have distinct features. Recently, in wheat and kiwifruit, circRNAs have been found to play important roles in plant dehydration, and immune response, respectively [11,12]. In tomato, circRNAs has been shown to participate in fruit ripening regulation, chilling responsive process, pigment accumulation, and the ethylene signaling pathway [13,14,15,16]. These recent advances have renewed our interest in this novel class of non-coding RNAs. Nevertheless, further work is needed to dissect the regulating mechanism of circRNAs in plants.

Potato, after maize, rice, and wheat, is the forth-largest food crop in the world [17]. However, its safe production is seriously threatened by *Pectobacterium carotovorum* subsp. *brasiliense* (*Pcb*), the emerging member of the soft rot *enterobacteriaceae* (SRE) species, which is the most important causal agent of potato blackleg and soft rot globally [18]. Previous studies have shown that non-coding RNAs (e.g., lincRNAs) play critical roles in regulating potato gene expression in response to *Pcb* infection [19]. However, to our knowledge, the role of circRNAs in potato-*Pcb* interaction is currently unclear. In this study, we focus on: (1) the identification and characterization of potato circRNAs in response to *Pcb* infection; (2) Gene ontology (GO) enrichment analysis of the parental genes and miRNAs targeted mRNAs of differentially expressed (DE) circRNAs to uncover their possible roles in regulating potato immune response; and (3) by using weighted gene co-expression network analysis (WGCNA) to infer the possible regulating networks among coding genes, circRNAs, and lincRNAs.

## 2. Results and Discussion

### 2.1. Genome-Wide Identification of circRNAs in Potato

**Figure 1:**
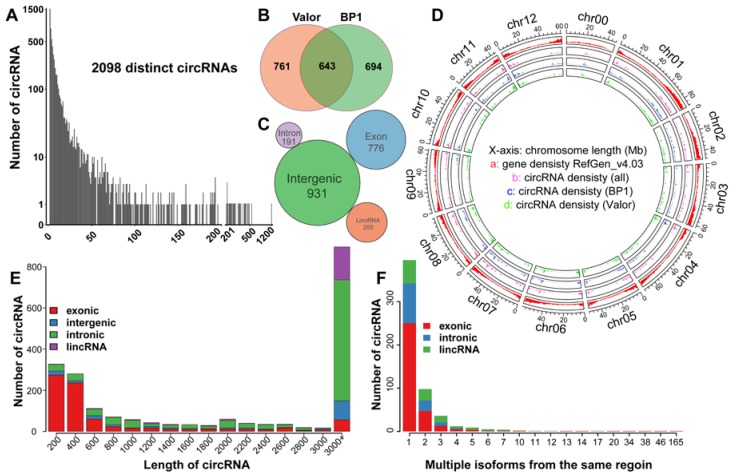
Characterization of potato circular RNAs (circRNAs). (**A**) Number of circRNAs and their supporting backsplicing reads. (**B**) Distribution of circRNAs in both cultivars. (**C**) CircRNAs genomic origin. (**D**) Distribution of circRNAs in each chromosome. (**E**) Distribution of circRNAs in different length ranges (e.g., 200 represents 0–200). (**F**) Summary of alternative circularization events.

Until now, circRNAs have been identified in multiple monocot and dicot plants [13]. However, our current understanding of the characteristic and biological roles of circRNAs in the potato-pathogen interaction remains limited. In this study, 13.8 billion 90 bp paired-end raw reads from two potato cultivars were used for circRNAs identification. Low quality reads were filtered and clean reads were mapped to reference genome by Burrows-Wheeler Aligner (BWA) (v0.7.15, mem -T 19), resulting in about 10.6 billion reads being uniquely mapped. The Sequence Alignment/Map (SAM) files of alignment were then scanned by circRNA identification tool CIRI2, which led to the identification of 76,882 junction reads. And 2098 circRNAs were finally detected in these thirty samples supported by at least two unique back-spliced reads, with 1404 in cultivar Valor and 1337 in cultivar BP1 (Tables S1 and S2). Among these identified circRNAs, 761 were specifically expressed in Valor, and 694 in BP1, respectively, suggesting that circRNAs are cultivar specific (Figure 1A,B).

### 2.2. The Characteristics of circRNA Abundance in Potato

Among the 2098 identified circRNAs, about half (931, 44.38%) were intergenic circRNAs, 776 (36.99%) were exonic circRNAs, 200 (9.53%) were lincRNA-derived circRNAs, and the remaining 191 (9.10%) were intronic circRNAs (Figure 1C). Similarly, in wheat and kiwifruit, the majority of the circRNAs were intergenic circRNAs (51% and 60.2%, respectively) [11,12]. However, in *Arabidopsis*, rice, and tomato, most of the identified circRNAs were found to be exonic circRNAs, suggesting that circRNA production is species-specific [10,13,14,20]. Regarding chromosome distribution, chromosome 9 produced most of the circRNAs, followed by chromosome 1 and 4 (Figure 1D). The length count analysis showed that most exonic circRNAs are shorter than 2000 nucleotides (nt), whereas most intergenic and lincRNA-derived circRNAs are longer than 2000 nt (Figure 1E, Table S1). Alternative circularization analysis identified 407 alternative backsplicing circularization events originating from 176 unique chromosome loci, including 79 from exon region, 59 from lincRNA region, and 38 from intron region. In total, 98 of 407 loci produced two different circRNA isoforms, 36 produced three distinct isoforms, 12 produced four isoforms, nine produced five isoforms, and one region produced one hundred and sixty-five distinct circRNAs isoforms (Figure 1F, Table S3).

### 2.3. CircRNAs Are Differentially Expressed in Response to Pcb Infection

**Figure 2:**
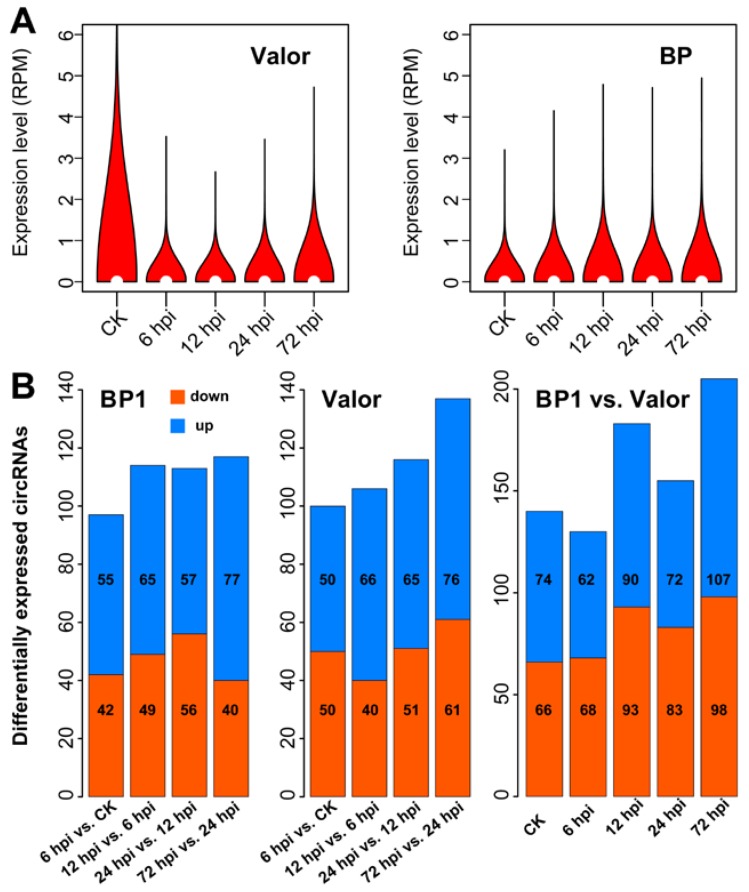
Differentially expressed circRNAs in response to potato-*Pectobacterium carotovorum* subsp. *brasiliense* (*Pcb*) infection. (**A**) Violin plot of relative abundance of circRNAs. Data are expressed as the RPM (reads per million mapped reads). (**B**) Number of differentially expressed circRNAs. vs. means versus.

These two potato cultivars, Valor (susceptible) and BP1 (disease tolerant), enabled us to identity the changes of circRNAs expression during infection and trace the underlying disease resistance-related circRNAs. Wilcoxon rank-sum test indicated that circRNAs are significantly down-regulated by *Pcb* infection in susceptible cultivar Valor (CK vs. 24 hpi, *p* = 0.04; CK vs. 72 hpi, *p* = 0.02; CK means control and hpi means hours post inoculation), but significantly up-regulated by *Pcb* infection in disease tolerant cultivar BP1 (CK vs. 12 hpi, *p* = 0.02; CK vs. 24 hpi, *p* = 0.05; CK vs. 72 hpi, *p* = 0.001), suggesting that they have different regulation patterns in two cultivars and likely play potential roles in immune response (Figure 2A). Among the 2098 circRNAs, 429 circRNAs had significant differential expression levels in response to *Pcb* infection, including 275 in BP1 and 287 in Valor, and 428 between BP1 and Valor (Figure 2B, Table S4).

### 2.4. CircRNAs Act as Putative Regulators of Parental Genes

**Figure 3:**
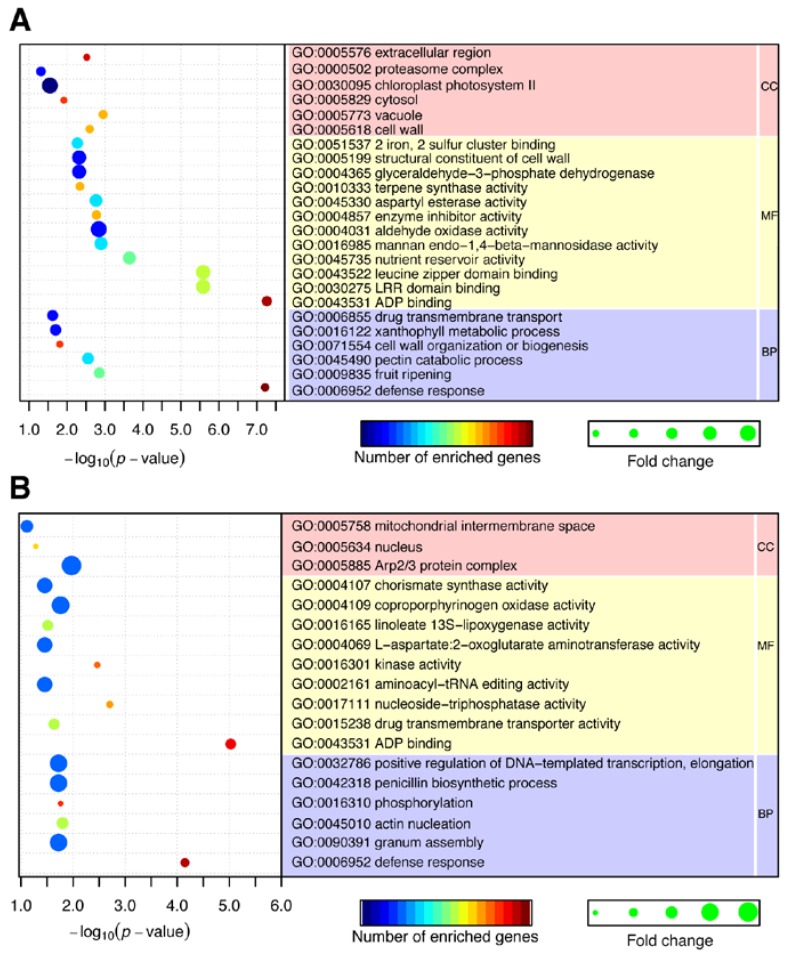
GO enrichment analysis of differentially expressed (DE) circRNAs. (**A**) Enriched by parental genes. (**B**) Enriched by miRNAs targeted mRNAs. GO, Gene Ontology; DE, differentially expressed; CC, cellular component; MF, molecular function; BP, biological process.

It was reported that circRNAs play important roles in controlling transcription by *cis*-regulating their parental genes [13]. In order to reveal the functions of DE circRNAs between susceptible and disease tolerant potato cultivars, the parental genes of significantly regulated circRNAs were predicted and annotated. GO enrichment analysis showed that these GO terms related to: (1) defense response (GO:0006952); (2) substance metabolism, including pectin catabolic process (GO:0045490), structural constituent of cell wall (GO:0005199), xanthophyll metabolic process (GO:0016122); and (3) energy metabolism, including chloroplast photosystem II (GO:0030095), ADP binding (GO:0043531), were specifically enriched. The enrichment result suggested the involvement of circRNAs in controlling potato responding to pathogen infection (Figure 3A, Table S5). These results are in accordance with those of previous studies, which suggested that plant resistance is a complex regulating network that involves in substance and energy metabolism, and defense process [21,22].

Cell wall metabolism is an important aspect in the basal disease resistance process [23]. In our study, we found that lots of circRNAs were produced by protein-coding genes that are involved in cell wall metabolism. For example, PGSC0003DMG400021603, annotated as extensin, can generate 165 circRNAs. And PGSC0003DMG400009783, annotated as vegetative cell wall protein, can give rise to 46 circRNA (Table S3). The high abundance of circRNAs derived from the cell wall related genes implies its biological importance. Consequently, it is plausible to assume that circRNAs play important roles during the interaction between potato and pathogen *Pcb*. Moreover, pectin catabolic process (GO:0045490) and xanthophyll metabolic process (GO:0016122), which have been reported playing roles in disease resistance [24,25], were also significantly enriched during GO analysis. In summary, these results suggest that circRNAs enriched in these GO terms associated with metabolism and defense may be involved in the regulation of disease resistance.

### 2.5. CircRNAs Act as Putative miRNA Sponges

Besides regulating parental genes, circRNAs have been proposed to play important roles in miRNA function and transcription regulation by acting as competing endogenous RNAs [13]. miRNAs had been reported to participate in lots of plant stress resistance processes through regulating target genes’ expression [26]. Obtaining insight into the miRNAs targeted by circRNAs will help us to further understand the functional importance of circRNAs. In this study, miRNAs sponged by DE circRNAs were used to predict the target mRNAs. As a result, 138 circRNAs (among the 470 DE circRNAs) were found to contain miRNA binding sites, suggesting that these circRNAs may function as miRNA sponges in potato. Target prediction showed that 120 miRNAs were the targets of these 138 circRNAs (Table S6). Furthermore, 608 mRNA targets of 96 miRNAs were predicted (Table S7). We then conducted GO analysis on these targeted mRNAs. Similar to GO analysis results in the parental genes of circRNAs, defense response (GO:0006952) was specifically enriched (Figure 3B, Table S8). Besides, phosphorylation (GO:0016310), ADP binding(GO:0043531), and kinase activity (GO:0016301) were also specifically enriched. Transcription regulations play important roles in plant response to environmental stresses, including biotic and abiotic stresses [27]. And it is well known that a large amount of predicted miRNA targets in potato are transcription factors (TFs) [28]. Similarly, in this study, many of the predicted miRNA targets are various TFs, including bZIP, WRKY, and ethylene-responsive TF (Table S7). Gu et al. [29] reported that stu-miRNA5303, the specific miRNA family of *Solanaceous* plants, possesses target genes that are functionally related to responsive to abiotic stress, metabolic enzymes, and uncharacterized proteins. Interestingly, in this study, stu-miRNA5303, was sponged by 151 circRNAs (Figure S1). Thus, we speculate that these 151 circRNAs may regulate the target genes of stu-miRNA5303 in potato immune response by sponging stu-miRNA5303 family members.

### 2.6. mRNA-CircRNA-LincRNA Co-Expression Networks

**Figure 4:**
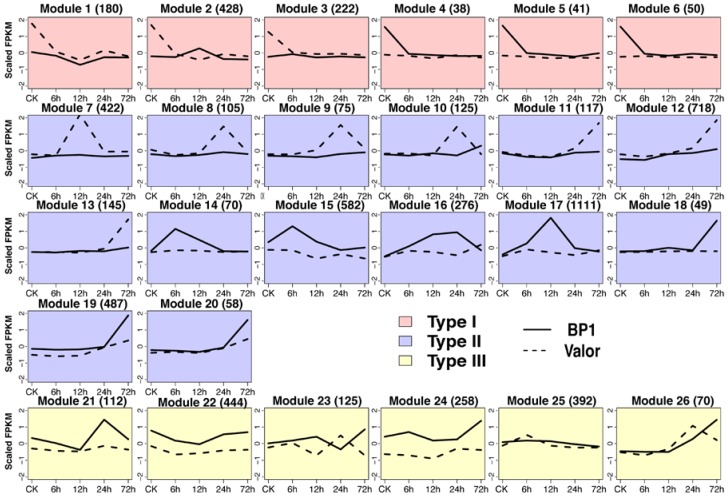
Changes in the expressions of the protein-coding genes, circRNAs and long intergenic noncoding RNAs (lincRNAs) within the modules over the stage of infections in two cultivars (dashed line, Valor; solid line, BP1). The different background colors represent for different module types divided by expression pattern.

WGCNA has been demonstrated to identify important genes associated with complex phenotypes and biological processes in plants [30]. It can identify clusters of highly co-expressed genes based on gene expression similarity [30]. To reveal the potential functions of circRNAs, a co-expression network analysis to 8928 DE genes was performed (Table S9). Totally, 26 modules which captured 6858 DE genes (5466 coding genes, 299 lincRNAs, and 1087 circRNAs) and corresponded to the clusters of 38 to 1111 highly co-expressed individuals, were identified (Table S10). These modules can be divided into three types based on their expression pattern (Figure 4).

Genes clustered in type I modules were characterized as suddenly down-regulated in either cultivar. In modules 1, 2, and 3, genes were down-regulated in Valor, but not in BP1 (Figure 4). Three modules enriched genes for photosynthesis and response to biotic stimulus (Table S11). For example, lots of receptor-like protein kinases (RLKs), which play important roles in activating plant innate immunity [31], were enriched in module 2. In module 2, RLKs were suddenly down-regulated in susceptible cultivar Valor, but slightly up-regulated in disease tolerant cultivar BP1 after 6 hpi, suggesting that rapid immune response in Valor were suppressed, whereas BP1 has a more rapidly response to the infection. In modules 4–6, genes that are functionally related to negative regulation of metabolism, were down-regulated in BP1, but not in Valor, which is consistent with disease tolerant phenotype of cultivar BP1 (Figure 4).

Genes clustered in 14 type II modules were characterized as only up-regulated in one cultivar, BP1 or Valor. Rapidly transcriptome reprograming is crucial for the immune response of host to pathogen infection [32]. Obviously, in BP1, genes clustered in modules (e.g., 14–17) were up-regulated immediately at 6 hpi, whereas, in Valor, genes clustered in modules (e.g., 8, 9, 10, and 11) were up-regulated after 24 hpi. The distinct regulating patterns indicated the different defense response of two cultivars. Specifically, in BP1, module 17 formed a large cluster containing 1111 individuals, and among them, 1045 genes are functionally related to defense response, such as numerous RLKs (receptor like kinases) (e.g., wall-associated kinases, WAK1; brassinosteroid insensitive 1-associated receptor kinase, BAK1. Table S12), l-phenylalanine metabolic process (GO:0006558), and cell wall thickening (GO:0052482), which were reported to have vital roles in plant growth and disease resistance (Figure S2, Table S9) [31,33,34]. Module 16 showed a similar expression pattern as 17, and clustered genes were functionally related to response to ethylene (GO:0009723). The production of ethylene (ET) was the resulting hallmarks of pattern-triggered immunity (PTI) [35], and ET signaling is required for oxidative burst contributing to plant immunity in PTI [36]. Cuticle provides a physical barrier against pathogens’ infection and therefore plays a basal role in disease resistance [37]. Module 15 contains genes functionally associated with cuticle development (GO:0042335) and cell wall organization (GO:0071555). In summary, these results indicated that the disease tolerance cultivar BP1 has a more quick and effective response to the pathogen infection, as these genes functionally related to cell wall, lignin synthesis, and ET were rapidly, robustly, and selectively transcriptionally reprogramed, which finally strengthen cell wall and launch PTI (pathogen-associated molecule patterns, PAMP triggered immunity) in cultivar BP1.

Genes clustered in type III modules showed disordered expression pattern. Nevertheless, interestingly, genes clustered in module 22 show higher expression level in BP1 than in Valor at each infection stage. These genes were functionally related to lignin metabolic process (GO:0009808), such as cellulose synthases and NAC (standing for no apical meristem (NAM), arabidopsis transcription activation factor (ATAF) and cup-shaped cotyledon (CUC)) domain-containing protein that are involved in regulation of secondary wall biosynthesis and cell death (Table S13) [38]. The cultivar-specific expression patterns suggested their possibly defense-related roles in enhancing resistance to *Pcb*.

Based on the co-expression relationship of circRNAs, lincRNAs, and coding genes, we inferred the potential functions of circRNAs in potato responsive to *Pcb* infections. Based on the co-expression analysis, we constructed a circRNA-centered (circRNA chr02:43604462|43622094) subnetwork, in which circRNA directly connected with acquired gene sets. In the subnetwork, circRNA chr02:43604462|43622094 was connected to many RLKs, TFs, and PRR (Pattern recognition receptor) coding-genes (Figure S3). These genes were GO enriched in l-phenylalanine metabolic process and cinnamic acid metabolic process that are closely related to cell wall composition. Besides, numerous lincRNAs were also connected with chr02:43604462|43622094, suggesting that lincRNAs may also participate in regulating the rapid transcriptional reprogramming involved in plant defense response. The networks analysis revealed the relationships among circRNAs, lincRNAs, and coding genes, and suggested the possible roles of circRNAs in transcriptome reprogramming during disease response.

### 2.7. Validation of the Identified circRNAs

**Figure 5:**
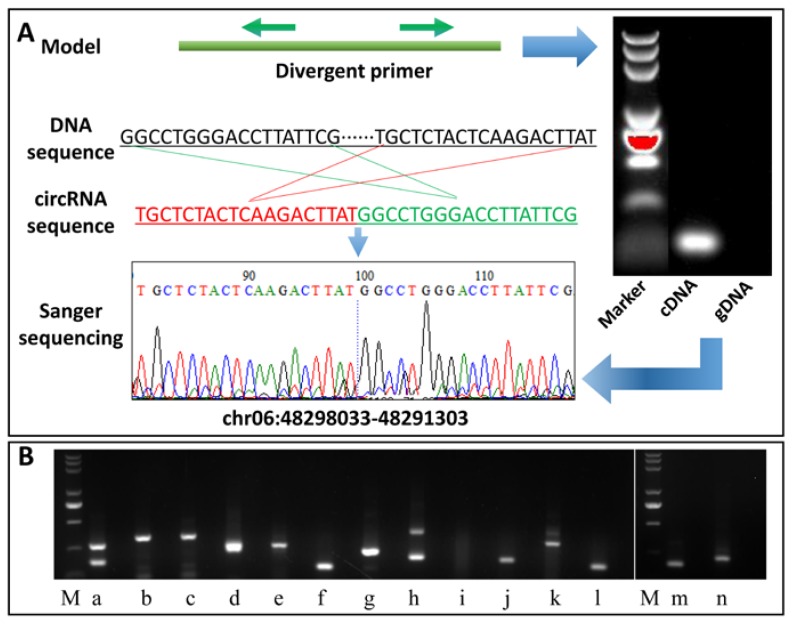
circRNAs validation by RT-PCR and Sanger sequencing. (**A**) A representative example chr06:48298033-48291303 and (**B**) thirteen other circRNAs were showed in the figure. Successfully validated circRNAs: a, chr02:31281485-31249653; b, chr04:5084821-5146050; c, chr04:5084836-5146050; d, chr01:58444823-58555059; e, chr01:79949288-79954313; f, chr04:63767513-63658440; g, chr12:57804736-57798617; h, chr06:46921692-46889410; j, chr11:9741794-9720571; k, chr11:40159197-40151022; l, chr06:48298033-48291303; m, chr09:15907865-15890843; n, chr05:5219204-5068751; not successfully validated circRNA: i, chr04:5137566-5146644 (M, DL2000 marker). Detailed information can be found in Table S14. Green arrows represent the PCR amplification orientation of divergent primers.

To validate the circRNAs identified from the circRNA-Seq analysis, reverse transcription RT-PCR and Sanger sequencing experiments were performed to 14 randomly selected circRNAs. A pair of divergent primers (Table S14) were designed for each circRNA, and both cDNA and gDNA (negative control) were used as template for PCR amplification (Figure 5). Finally, among the 14 circRNAs, 13 were successfully confirmed (92.86%), suggesting the reliability of our circRNAs identification result (Table S14), as comparing to kiwifruit (68/80, 85.00%), tomato (8/11, 72.73%), and rice (10/18, 55.56%), much higher rate of circRNAs were successfully validated in our study [10,12,13].

## 3. Materials and Methods

### 3.1. Potato RNA-seq Data Sets

Raw reads of RNA-seq and raw counts of lincRNAs were downloaded from the NCBI (GSE74871). The dataset includes five time points (CK, 6 hpi, 12 hpi, 24 hpi, and 72 hpi, and each time point with three replications) from two potato cultivars stem tissues (Valor, susceptible; BP1, disease tolerant) infected by *Pcb* [19]. Raw reads were trimmed by Trimmomatic (v0.36) for removing low quality reads [39]. Trimmed reads were aligned to potato genome [17] using TopHat2 (v2.0.13, -library-type fr-firststrand -G -i 10 -l 15,000) [40]. The DE genes identification were performed by Cuffdiff (v2.2.1) and DESeq2 (v1.16.1) using the cut-off criteria of a false discovery rate adjusted *p*-value ≤ 0.05 and |log_2_(foldchange)| ≥ 1, then selected the overlap of these two results [40,41]. The GO annotation and GO enrichment were performed by Blast2go (e-value 1 × 10^5^.) and top GO with default parameters, respectively [42,43]. All raw counts of lincRNAs were normalized by DESeq2 [41]. All analysis pipelines were built by snakemake (v4.0.0) [44].

### 3.2. Identification and Expression Analysis of Circular RNAs

Trimmed reads were mapped to potato genome by BWA (v0.7.15, mem -T 19) [45]. The SAM file of alignment was then inspected using CIRI2 (v2.0.5) to identity circRNAs with the default parameters [46]. To compare the expression of circRNAs across time and taxa, we calculated the counts of backspliced reads for each circRNA and normalized the expression levels by comparing counts with mapped reads in corresponding sample (defined as reads per million mapped reads, RPM). DE circRNAs were defined as |log_2_(foldchanges)| ≥ 1.

### 3.3. Prediction of miRNA Target

The FASTA formatted circRNA sequences were used as queries and input into psRNAtarget (http://plantgrn.noble.org/psRNATarget/) to predict their sponging miRNAs [47]. Then miRNAs sponged by circRNAs were used as queries to predict their target mRNAs using TargetFinder through searching potato coding genes assembled by cufflinks [48].

### 3.4. Gene Co-Expression Network Analysis

The co-expression network analysis was performed to group DE genes (including coding genes, lincRNAs, and circRNAs) into modules using R package WGCNA (v1.51) [49]. In brief, we applied an optimal power function (β) as 20 to balancing the scale-free property of the co-expression network and sparsity of connection between genes. And the cutreeDynamic function was performed with parameters as deepSplit = 2, pamRespectDendro = F, minClusterSize = 30, and finally using the mergeCloseModules with cut Height set to 0.25 to merge these highly correlated clusters. Modules were visualized using Cytoscape (v3.4.0), setting the layout with edge-weighted spring embedded layout [50].

### 3.5. Reverse Transcription (RT)-PCR

To validate the identified circRNAs in potato, total RNA was extracted using Plant RNA Kit (Omega, London, UK) and cDNA was synthesized by reverse transcription using RevertAid First Strand cDNA Synthesis Kit (Thermo Scientific, Waltham, MA, USA). The divergent PCR primers were designed using the “out-facing” strategy to exclude linear mRNA from amplification (synthesized by Sangon Biotech Co., Ltd., Shanghai, China; Table S14). PCR products were validated by Sanger sequencing.

## 4. Conclusions and Perspectives

CircRNAs constitute a family of transcripts with unique structures and functions that are still largely unknown, especially in plants. In this study, through the expression patterns analysis and gene function annotation, we speculated that circRNA may participate in the regulation of potato immune response by regulating parental genes as well as acting as putative miRNA sponges. As GO enrichment showed that circRNAs were predicted to be involved in defense response (GO:0006952), cell wall (GO:0005199), ADP binding (GO:0043531), phosphorylation (GO:0016310), and kinase activity (GO:0016301). Furthermore, WGCNA found circRNAs being closely related with mRNAs and lincRNAs. And together they were cultivar-specifically regulated to strengthen immune response of potato to *Pcb* infection, implying the roles of circRNAs in reprogramming disease responsive transcriptome. The results reported in the present study lay a foundation for further studies of the roles that circRNAs play in potato immune response. The precise functions of circRNA in potato immune response need to be experimentally tested in the following researches.

## Supplementary Materials

Supplementary materials can be found at www.mdpi.com/1422-0067/19/1/71/s1.

## Abbreviations

*Pcb*	*Pectobacterium carotovorum* subsp. *brasiliense*
circRNAs	Circular RNAs
miRNA	MicroRNA
hpi	Hours post-infection
WGCNA	Weighted gene co-expression network analysis
SRE	Soft rot *Enterobacteriaceae*
GO	Gene Ontology
WAK	Wall-associated kinases
BAK1	Brassinosteroid insensitive 1-associated receptor kinase
PTI	Pathogen-associated molecule patterns triggered immunity
ET	Ethylene
RLK	Receptor-like protein kinase
TFs	Transcription factors
MF	Molecular function
BP	Biological process
CC	Cellular component
