# Supplementary material for: Transcriptome-Wide Identification and Characterization of Potato Circular RNAs in Response to Pectobacterium carotovorum Subspecies brasiliense Infection
Source: Int J Mol Sci. 2017 Dec 27;19(1):71. doi: 10.3390/ijms19010071 (PMC5796021; doi:10.3390/ijms19010071)
Supplement: Supplementary file 1 [file ijms-19-00071-s001.zip › Supplementary caption.docx]

Figure S1 Potato miRNA families sponged by circRNAs;

Figure S2 A representative module network (module 17);

Figure S3 A circRNA-centered subnetwork;

Table S1 Detailed information of detected circRNAs;

Table S2 Sequences of detected circRNAs;

Table S3 Multiple circRNA isoforms from the same region;

Table S4 Significant DE circRNAs detected among pair-wised comparisons;

Table S5 GO enrichment of parental genes of significant DE circRNAs;

Table S6 miRNAs sponged by circRNAs;

Table S7 miRNA targeted mRNAs and their annotations;

Table S8 GO enrichment of miRNA targeted mRNAs of significant DE circRNAs;

Table S9 Significant DE genes identified by cuffdiff and DESeq2;

Table S10 Significant DE genes used for co-expression networks analysis;

Table S11 GO enrichment results of genes clustered in type1 modules;

Table S12 GO enrichment results of three type2 modules (15–17);

Table S13 GO enrichment results of module 22;

Table S14 Divergent primers used for circRNA validation.tABLE
