# Supplementary figures and images for: Transcriptome-Wide Identification and Characterization of Potato Circular RNAs in Response to Pectobacterium carotovorum Subspecies brasiliense Infection
Source: Int J Mol Sci. 2017 Dec 27;19(1):71. doi: 10.3390/ijms19010071 (PMC5796021; doi:10.3390/ijms19010071)

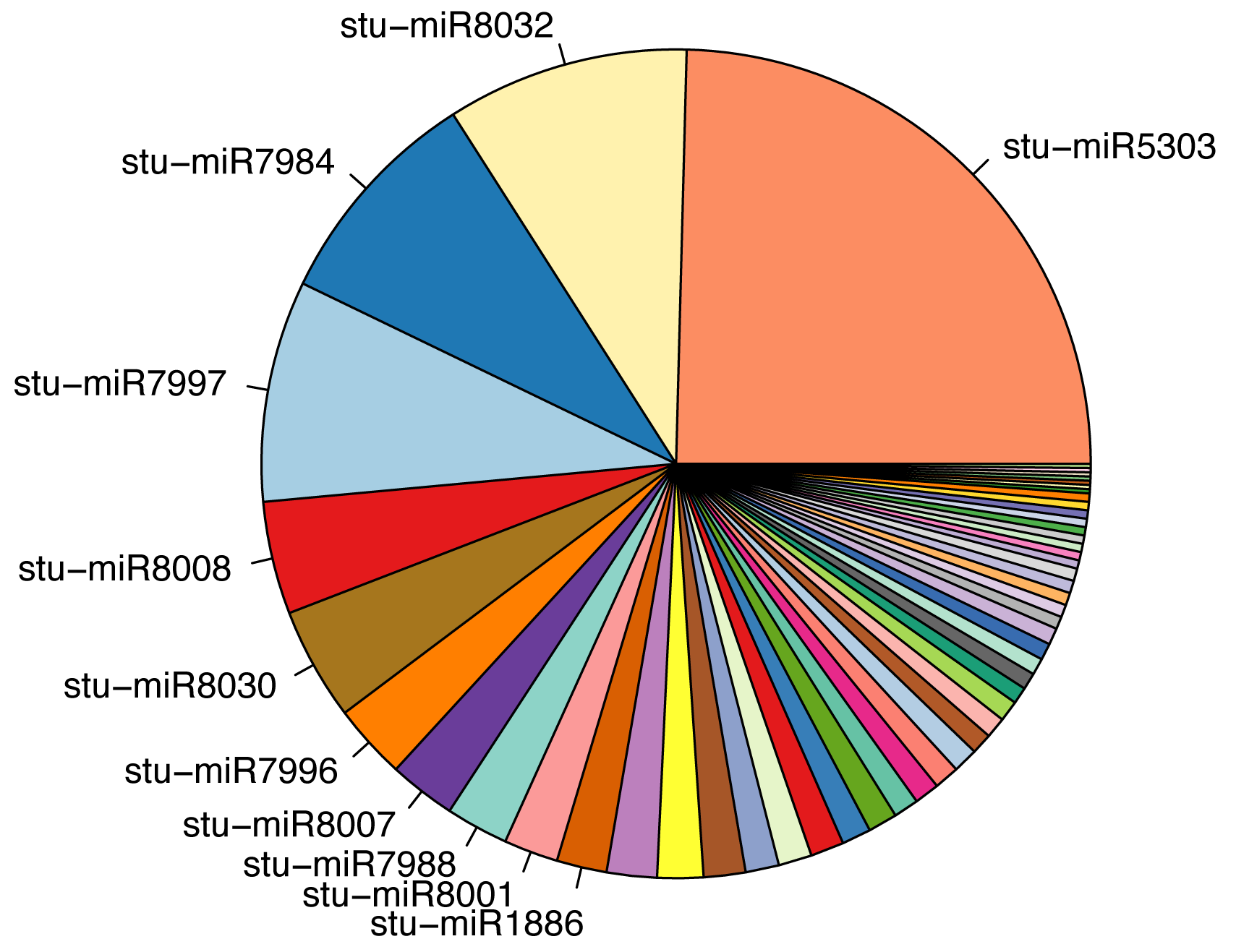

Supplement: Supplementary file 1 [file ijms-19-00071-s001.zip › Figure S1 Potato miRNA families sponged by circRNAs.tif]

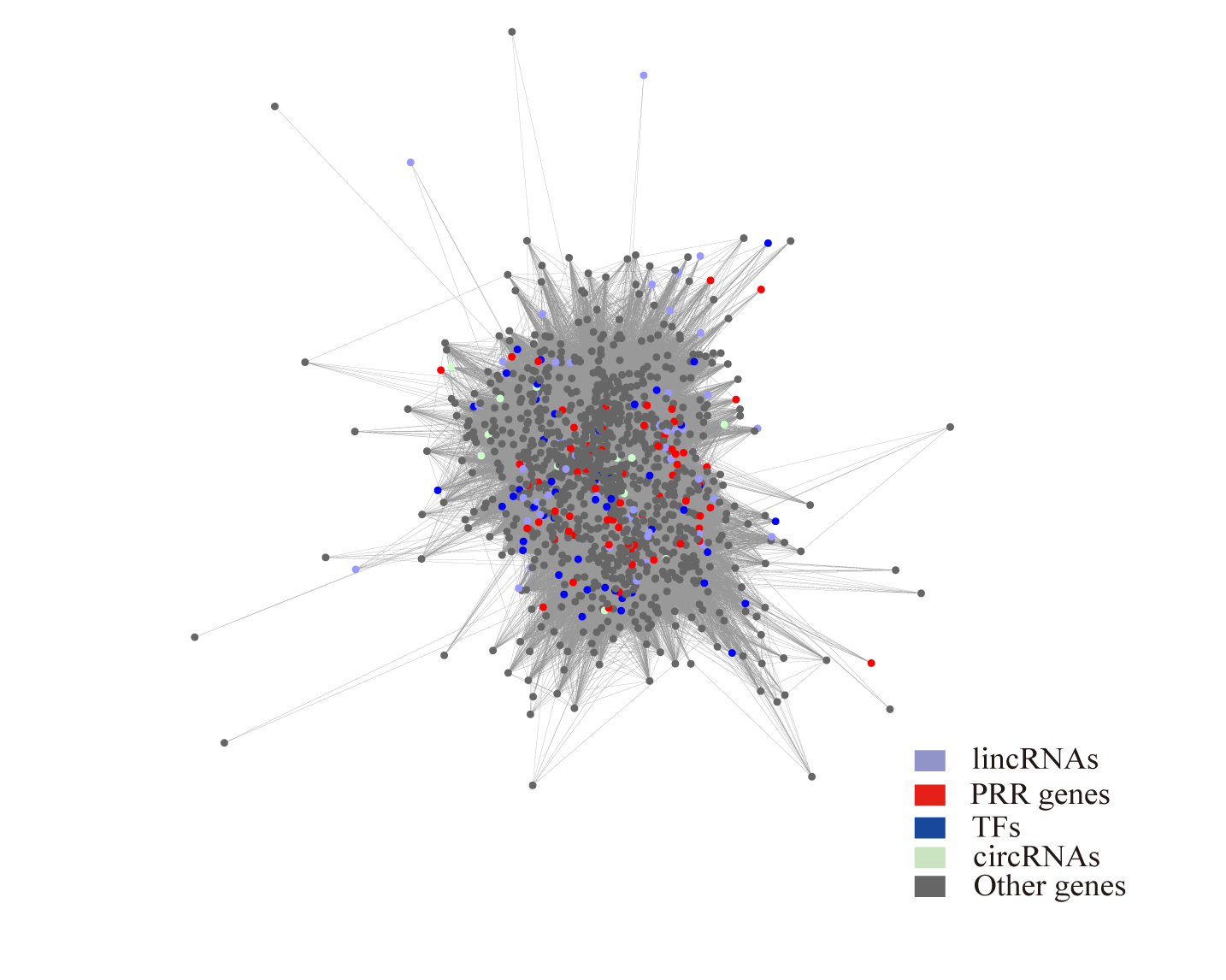

Supplement: Supplementary file 1 [file ijms-19-00071-s001.zip › Figure S2 A representative module network (green).tif]

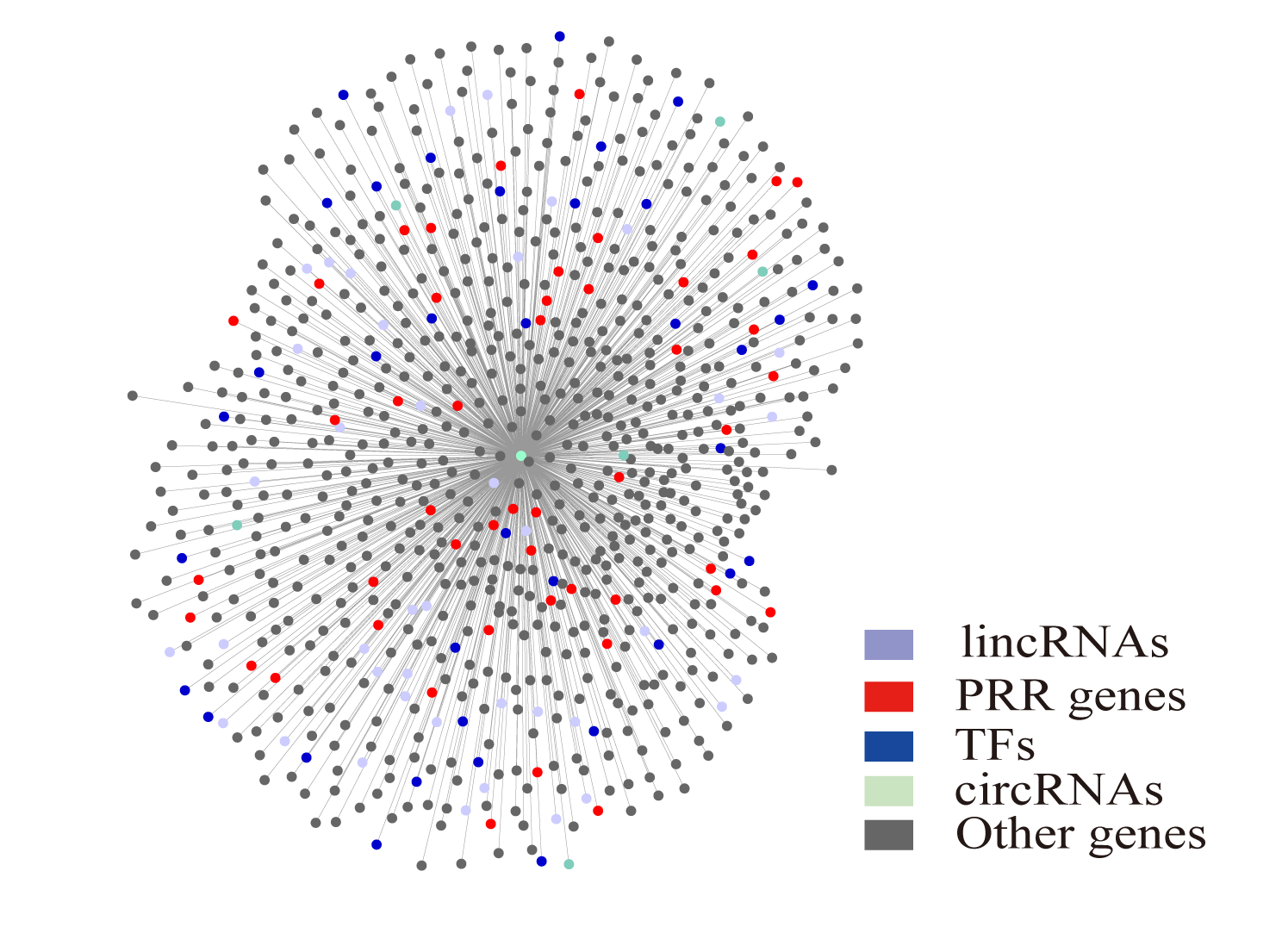

Supplement: Supplementary file 1 [file ijms-19-00071-s001.zip › Figure S3 A circRNA-centered subnetwork.tif]
